# Integrated tunable green light source on silicon nitride

**DOI:** 10.1038/s41377-026-02222-8

**Published:** 2026-02-28

**Authors:** Gang Wang, Ozan Yakar, Xinru Ji, Marco Clementi, Ji Zhou, Christian Lafforgue, Jiaye Wu, Jianqi Hu, Tobias J. Kippenberg, Camille-Sophie Brès

**Affiliations:** 1 École Polytechnique Fédérale de Lausanne, Photonic Systems Laboratory (PHOSL), Lausanne, Switzerland; 2 École Polytechnique Fédérale de Lausanne, Laboratory of Photonics and Quantum Measurements (LPQM), Lausanne, Switzerland; 3 Dipartimento di Fisica “A. Volta”, Universitá di Pavia, Pavia, Italy

**Keywords:** Integrated optics, Nonlinear optics

## Abstract

Integrated green light sources are essential for telecommunications and quantum applications, while the performance of current on-chip green light generation is still limited in power and tunability. In this work, we demonstrate green light generation in silicon nitride microresonators using photo-induced second-order nonlinearities, achieving up to 3.5 mW green power via second-harmonic generation and densely tunable over a 29 nm range. In addition, we report milliwatt-level all-optical poling (AOP) threshold, allowing for amplifier-free continuous-wave AOP. Furthermore, we demonstrate non-cascaded sum-frequency generation, leveraging the combination of AOP and simultaneous coherent frequency combs generation at 1 *μ*m. Such comb-assisted AOP enables switching of the green light generation over an 11 nm range while maintaining the pump within a single resonance. The combination of such highly efficient photo-induced nonlinearity and multi-wavelength AOP enables the realization of low-threshold, high-power, widely-tunable on-chip green sources.

## Introduction

Green light sources, typically defined by emission wavelengths within the 510–560 nm range, have wide applications in fields such as quantum photonics^1–3^, material processing^4^, and underwater communication^5^. While semiconductor lasers are widely available in the blue and red wavelength regions, efficient green laser sources are difficult to produce due to the low internal quantum efficiency^6^ and are often limited in tunability. As such, nonlinear conversion is the standard approach to generate green light, which has been widely adopted in tabletop systems based on crystals.

Recently, progress in photonic integration has led to the realization of compact and scalable on-chip green light sources based on second-order (*χ*^(2)^) and third-order (*χ*^(3)^) nonlinearities, leveraging frequency conversion processes such as third-harmonic generation (THG)^7–12^, optical parametric oscillation (OPO)^13–15^, sum-frequency generation (SFG)^16–18^, and second-harmonic generation (SHG)^19,20^. On-chip green light via THG has been generated in silicon photonic crystal waveguides^7^, silicon nitride (Si_3_N_4_) waveguides and microrings^8–10^, AlN microrings^11^, and composite Si_3_N_4_/AlN microrings^12^ where up to 49 *μ*W at 514 nm was achieved. *χ*^(3)^ OPO-based green sources in Si_3_N_4_ microrings pumped near 750 nm^13–15,21^ have shown significantly improved tunability over the visible spectrum, achieving a maximum on-chip green power of 100 *μ*W^15^.

Frequency conversion via *χ*^(2)^ nonlinearity is generally a more efficient approach. Cascaded SHG and SFG with a telecom pump has been exploited in periodically poled thin-film lithium niobate microring^16^ reaching up to 334 *μ*W at 520 nm, yet the efficient generation has been restricted to a single resonance. SHG in peridiodically poled lithium tantalate waveguide^19^ resulted in 1.87 mW at 532 nm, with a 0.40 nm generation bandwidth. In amorphous materials, despite lacking intrinsic *χ*^(2)^ nonlinearity, an effective *χ*^(2)^ can be induced through all-optical poling (AOP), firstly observed in silica fibers^22–25^ then in Si_3_N_4_ waveguides and microresonators^17,18,20,26–31^. Based on multi-photon absorption interference involving three photons with angular frequencies *ω*_1,2,3_ (*ω*_3_ = *ω*_1_ + *ω*_2_), a directional photocurrent *j*_ph_ is generated via the coherent photogalvanic effect (PGE), promoting the build-up of an electrostatic field *E*_DC_. The steady state of *E*_DC_ can be expressed as *E*_DC_ = − *j*_ph_/*σ*, where *j*_ph_ and photoconductivity *σ* are functions of *ω*_1,2,3_^17^. The static electric field induced by the AOP process exhibits a periodic distribution along the propagation axis with a wavevector Δ*k* = *k*_3_ − *k*_1_ − *k*_2_ = 2*π*/*Λ*, where *k*_1,2,3_ represent the wavevectors of *ω*_1,2,3_ and *Λ* is the spatial period of *E*_DC_. As such, an equivalent periodically modulated second-order nonlinearity ${\chi }_{{\rm{eff}}}^{(2)}=3{\chi }^{(3)}{E}_{{\rm{DC}}}$ is inscribed, satisfying the first-order quasi-phase-matching (QPM) condition for the *χ*^(2)^ frequency conversion processes satisfying *ω*_3_ = *ω*_1_ + *ω*_2_. While most studies have been carried out in the 1550 nm telecom band generating a near-infrared second-harmonic (SH), green light generation from a 1 *μ*m pulsed pump in an optically poled Si_3_N_4_ waveguide has also been demonstrated^20^. In addition, cascaded SFG after photo-induced SHG from a telecom pump have also been shown in Si_3_N_4_ waveguides and microresonators^17,18^. So far, the green light generation in Si_3_N_4_ has been limited to a sub-mW level^10,14,20^, and the trade-off between power and tunability is still challenging to solve.

In this work, we demonstrate an integrated green light source based on AOP in two Si_3_N_4_ microresonators, achieving up to 3.5-mW green power and dense tunability over a 29 nm range. Furthermore, we report the AOP threshold as low as 4.5 mW of pump power required to trigger continuous-wave (CW) AOP process, which overcomes the need for optical amplifiers. By leveraging the anomalous dispersion of our microrings at 1 *μ*m, we also investigate the interactions of photo-induced *χ*^(2)^ and *χ*^(3)^-based frequency comb generation. We show that comb-assisted AOP allows for non-cascaded SFG, which is widely switchable over a range of 11 nm by slight pump detuning in a single pumped resonance. These results advance on-chip efficient green light sources that are low-threshold, high-power and widely tunable.

## Results

**Fig. 1:**
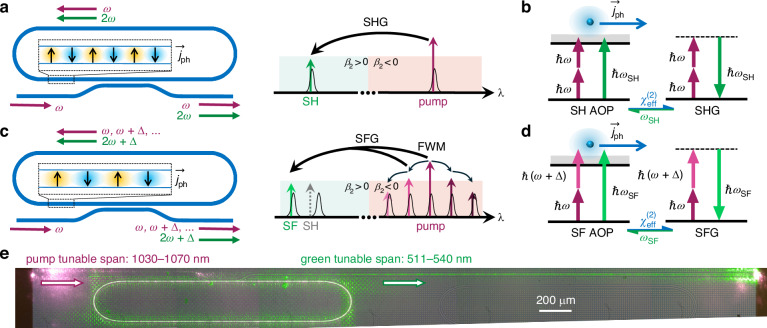
All-optical-poling-enabled green light generation in Si_3_N_4_ microresonators **a** Schematic of SHG in the Si_3_N_4_ microresonator when a doubly resonant condition is met. The dashed box shows the zoomed-in view of the optically induced photocurrent distribution. Black Lorentzian curves in the right figure represent resonances of the microresonator. **b** Illustration of SH-AOP-induced photocurrent generation and energy conservation for SHG. The FH and SH waves collectively contribute to the generation of a directed photocurrent through multi-photon absorption interference, thereby inscribing the built-in periodic electric field which further interacts with FH to generate SH. **c** Schematic of SFG in the Si_3_N_4_ microresonator when triply resonant condition is selectively met among two comb lines and the SF signal. At pump frequency the waveguide exhibits anomalous dispersion, allowing the injected pump at *ω* frequency to be converted into multiple comb lines with a frequency difference of Δ through cascaded four-wave mixing. SF AOP can generate its own *χ*^(2)^ grating different from the SH gratings, as shown in the dashed box. **d** Illustration of SF-AOP-induced photocurrent generation and the energy conservation for SFG. **e** Microscope image of the device under operation. Green light is generated in the ring and out-coupled to the right. With 1030--1070 nm pump, green light with a wavelength of 511--540 nm can be densely achieved. *β*_2_, second-order dispersion coefficient

The mechanism for green light generation in our microresonator is illustrated in Fig. 1, where the coherent PGE and four-wave mixing (FWM) play key roles. Two Si_3_N_4_ racetrack microresonators are used in our study. Both feature identical waveguide cross-sections of 1.3 × 0.9 *μ*m^2^ but differ by the coupling gap of 494 nm or 567 nm. In the pump wavelength (fundamental harmonic - FH) range around 1 *μ*m, the resonator exhibits anomalous dispersion for the fundamental mode. In the SH wavelength range, the fundamental mode and all higher-order modes identified in our poling process exhibit normal dispersion. Details on the device layout and dispersion calculations are given in Supplementary Note 1 and shown in Fig. S1.

Figure 1a–b describes the photo-induced SHG process initiated by AOP. A tunable CW 1-*μ*m pump (1030–1070 nm) is injected into the bus waveguide and couples to the resonator through a directional coupler as the fundamental TE mode. When the FH and SH are both in resonance, i.e., doubly resonant, SHG can be achieved through the following positive feedback in Fig. 1b, until an equilibrium is achieved between the photogalvanic current and the photoconductivity-induced drift current^17,30^. The photocurrent ${\vec{j}}_{{\rm{ph}}}$ and the electric field ${\vec{E}}_{{\rm{DC}}}$ have a spatial distribution as shown in the zoomed view of Fig. 1a. Given that the waveguide is multimode in the SH band, AOP can occur via the interaction between the fundamental pump mode and SH higher-order transverse modes, resulting in an adaptation of the grating period as well as its geometrical shape^29^.

Figure 1c–d depicts the interactions between FWM and photo-induced SFG processes. When a triply resonant condition among two comb lines and the SF signal is met, for example between *ω*, *ω* + Δ and 2*ω* + Δ, this non-cascaded SFG can be realized similar to the aforementioned SHG process, regardless of the dispersion regime in the green band. QPM grating inscription and SFG can be enhanced mutually through a positive feedback as shown in Fig. 1d. This comb-mediated AOP leads to self-sustained SFG, which indicates the SFG writes its own grating instead of relying on any pre-inscribed gratings. This process effectively addresses the frequency limitations encountered in both degenerate and cascaded SFG methods, resulting in improved tunability. Figure 1e shows a micrograph of the device under operation, where the green light is efficiently generated in the cavity and coupled out to the bus waveguide.

### High-power and reconfigurable on-chip green source

First, AOP for SHG is carried out in a Si_3_N_4_ racetrack microresonator with a free spectral range (FSR) of 50 GHz and a 494-nm gap between the ring and the bus. Detailed characterization, experimental setup, and mode profiles of participating SH modes (defined as SH1 to SH4) are provided in the Supplementary Note 1. The loaded quality factor (Q) of a typical pump resonance is measured to be 0.8 × 10^6^ as shown in Fig. S2, with intrinsic Q of 12.8 × 10^6^, overcoupled at the pump.

**Fig. 2:**
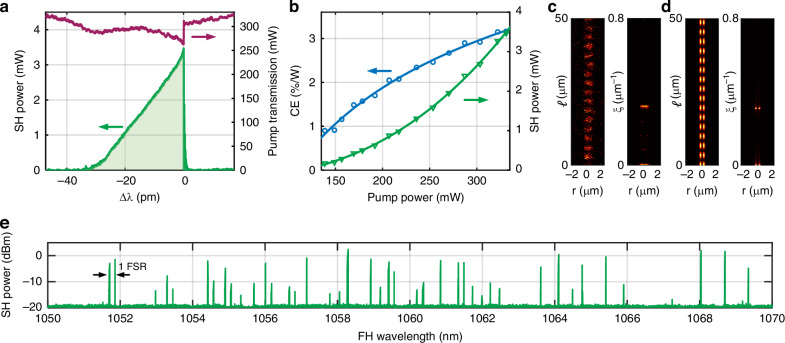
Reconfigurable high-power green light generation. **a** Nonlinear resonance sweep where the green and purple curves represent the on-chip SH power and the pump transmission. **b** SH conversion efficiency and output power as a function of pump power. **c** Experimental two-photon microscope image and calculated spatial frequency distribution, which are in good agreement with **d** simulations involving the interaction between the fundamental FH mode and SH2. *ℓ*, propagation distance; *r*, radial distance; *ξ*, spatial frequency. **e** Broadband SHG via reconfigurable AOP. Approximately 40% of resonances can achieve AOP. FSR: free spectral range

Figure 2a presents the optimized fast resonance sweep with a speed of 3.7 nm/s at 1068 nm under a pump power of 25.2 dBm in the bus waveguide, where the on-chip SH power reaches a maximum of 5.4 dBm (3.5 mW). The pump transmission exhibits an atypical thermal triangle shape, attributed to the Fano lineshape of the resonance^32^. The change in SH power as a function of detuning depends on the thermal shift speed difference between the SH and FH band, and on the effective SH detuning^30^, which for this particular AOP event result in monotonic increase in power. The conversion efficiency (CE = ${P}_{{\rm{SH}}}/{P}_{{\rm{pump}}}^{2}$) and the on-chip SH power as a function of the on-chip pump power are shown in Fig. 2b, reaching 3.2 %/W and 3.5 mW, respectively.

The inscribed grating can stay inside the Si_3_N_4_ microring after AOP^27^, which can be measured by two-photon microscropy (TPM)^27,29^. For the resonance under test, the TPM pattern reveals the spatial distribution and associated spatial frequency shown in Fig. 2c, which aligns simulation considering the interaction between the transverse mode pair FH-SH2 (*ξ* = 0.28 *μ*m^−1^), as shown in 2d. Other TPM patterns in different resonances, written by other transverse mode pairs FH and SH modes from SH1 to SH4 are also observed (Supplementary Note 3).

AOP occurs for a multitude of resonances when a single pump wavelength sweep is performed from 1050 to 1070 nm. Figure 2e illustrates the reconfigurable SHG under a stabilized temperature of 51°C. The pump power is 24.8 dBm in the bus waveguide and the wavelength of the pump laser is swept with a speed of 0.01 nm/s. Among all 105 pumped resonances, 42 exhibited detectable SHG. Note that there is no comb generation at the pump wavelength, as the pump power is below threshold. A detailed wavelength scan conducted within the 1060–1070 nm range reveals that approximately 63% of resonances yield green light output, as illustrated in Fig. S4.

### Low-AOP-threshold on-chip green source

The AOP process is known to exhibit a poling threshold^30^, which at 1550 nm telecom wavelength has been observed to decrease with the finesse of the resonator. A low threshold is particularly desirable for fully integrated devices such as the self-injection locking SHG laser source^33,34^, as it would eliminate the necessity for a table-top amplifier, thereby reducing operational complexity and enabling independent reconfiguration or maintenance of effective *χ*^(2)^ gratings. AOP threshold for devices operating at C band of few mW was measured in perfect-phase-matching high-finesse large-FSR Si_3_N_4_ microrings^28,35^. However, small rings are significantly restricted in AOP tunability while larger microresonators suffer from a significantly increased threshold at telecom wavelengths.

**Fig. 3:**
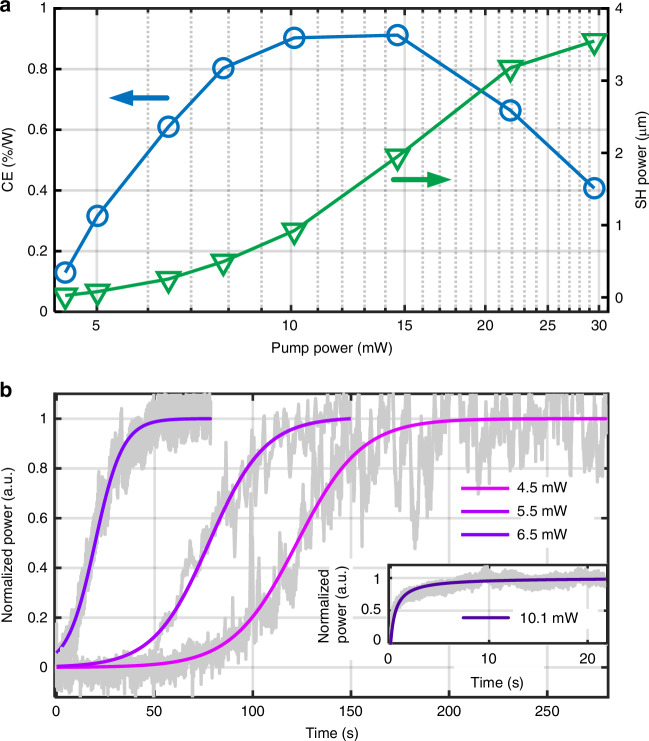
Low-threshold AOP with milliwatt-level pump. **a** Conversion efficiency and output power of SH as a function of pump power. The threshold for AOP and harmonic generation is measured at 4.5 mW. **b** Growth of generated SH power over time under low pump powers. Gray traces: experimental data; purple curves: sigmoid fits. The growing SH power proves the capability of self-assisted low-threshold AOP. Inset: rapid growth of SH power under a pump power of 10.1 mW

The physical principles of AOP and of the coherent PGE, suggest that operating the system at shorter pump wavelengths offers a significant opportunity for lowering the AOP threshold. The coherent PGE relies on charge excitation, where shorter wavelength light is more prone to be absorbed and excite the charges. Therefore, the photogalvanic coefficient at 1 μm should be higher than that at telecom wavelength, enabling low-threshold AOP even in small FSR rings. This property could overcome the need for an amplifier and facilitate fully on-chip reconfigurable AOP. We investigated the onset of AOP in our 50 GHz ring with a gap of 567 nm exhibiting a loaded Q of 1.8 × 10^6^ (see detailed characterization in Supplementary Note 1). The results are shown in Fig. 3a, plotting the measured variations in the CE (blue) and the SH power (green) as a function of the pump power in the bus waveguide. Despite a significantly smaller finesse (320) than that of the previous demonstration^28^ (~ 3.1 × 10^3^), the AOP threshold in this work is still lower and is estimated at 4.5 mW in the bus waveguide. The stronger PGE at short wavelength contributes to the lowering of the poling threshold. Beyond that value, the CE increases steadily until the pump power reaches approximately 15 mW, after which it starts to decrease. The reduction in CE corresponds to a decrease in the internal electric field strength, potentially caused by the bleaching of the electric field resulting from exposure to high-power, high-frequency light^17^.

To confirm that low-power green light generation is indeed self-assisted rather than the reading of a pre-written gratings, the measured growth of SH power as a function of time at low pump powers (4.5, 5.0, 6.5 mW) is illustrated in Fig. 3b, normalized to the maximum power obtained at each case. The gray and colored curves show the measured and sigmoid-fit SH power. Any pre-existing grating was erased with incoherent broadband light and verified via TPM, as also corroborated by the zero initial SH power in Fig. 3b. At a pump power of 5 mW, the growing trend indicated AOP and the half-rise time of SH power from the sigmoid fit is approximately 78 s. When the power in the bus waveguide increases to 6.5 mW, the poling time decreases to 18 s. At 10.1 mW, the half-rise time is less than 1 second as shown in the inset of Fig. 3b, with considerable CE. The observed increase in SH power deviates from the sigmoid approximation model, likely due to the increased nonlinear and thermal effects associated with higher input power levels. A 10-mW pump power represents a readily achievable threshold for on-chip lasers, demonstrating the capability of this microresonator device to facilitate fully integrated on-chip AOP. We can note that the previous device with a gap of 494 nm showed a threshold of 139 mW, attributed to the low finesse of the involved resonance.

### Frequency-comb-mediated AOP

In a microresonator with anomalous dispersion, Kerr combs such as primary combs, modulation instability (MI) combs, and different types of solitons states can be realized. The primary combs and solitons are coherent, namely they exhibit a specific phase relation between comb lines, while the noise-driven MI combs are incoherent^36,37^. Coherent combs can write and sustain QPM gratings^18,20,27^, while incoherent combs, due to their high power, high phase noise, and complex modes involved, could contribute to the erasure of gratings. Here, we demonstrate the poling and erasure of the gratings in a Si_3_N_4_ microring based on coherent and incoherent Kerr combs.

**Fig. 4:**
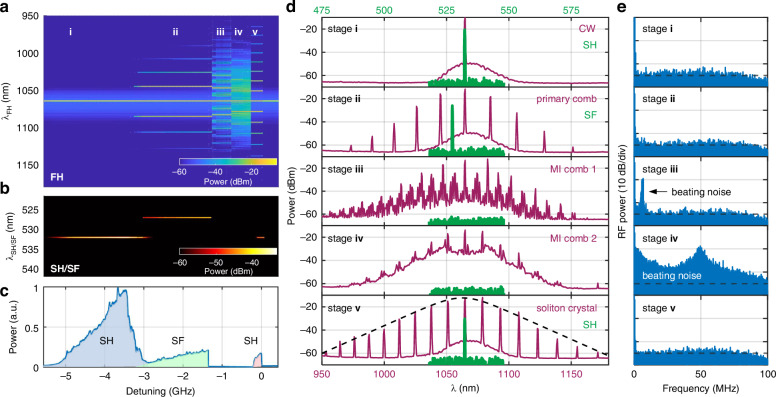
Kerr-comb assisted all-optical poling and erasing. **a**–**c** Evolution of FH spectra, green spectra, and green output power in a single linear resonance sweep. FH experiences five stages, three of which are coherent and facilitate AOP while the other two lead to grating erasure. The corresponding spectra of FH and SH/SF in five stages are shown and marked in **d**. **e** Measurement of beating noise in a 100 MHz span. The noise in stage **iii** and **iv** shows the beating signal of incoherent MI combs

During a single resonance sweep from blue to red detuning under a pump power of 23 dBm, we measure the optical spectrum in both the FH and SH band, as well as the radio frequency (RF) signals from the 100-MHz near-infrared photodetector at the output of our device. Figure 4a shows the evolution of the attenuated FH spectra recorded by the optical spectrum analyzer (OSA), where five stages appear, including CW, primary comb, first and second MI combs, and soliton crystal. Corresponding to the five stages of FH, green signals are observed and marked in Fig. 4b (OSA spectra) and Fig. 4c (green light power). Figure 4d–e display the detailed overlayed dual-axis FH and SH spectra, as well as low-frequency beating noise of the FH signal of these five stages.

In stage **i**, the FH is a 1064.6-nm CW as shown in Fig. 4d. A green signal at 532.3 nm gradually appears, corresponding to SHG, indicating that a doubly resonant condition is met. We can see in Fig. 4d that the SH signal is precisely located at half the wavelength of the FH. Figure 4e shows the noise floor of FH detected by a 100-MHz photodetector.

With the FH wavelength approaching the effective resonance wavelength (detuning approaching zero), the coupled FH power increases and modulation instability gain is triggered, enabling primary comb generation indicated in stage **ii**. Correspondingly, the SH signal gradually diminishes during the primary comb generation process. As the SH at 532.3 nm decreases, a SF signal at 527.3 nm gradually replaces the SH, indicating that a triply resonant condition for SFG is met. In the early stage of primary comb generation, SH and SF exist concurrently for a short range until SH completely disappears, suggesting that the SH grating is gradually erased while the SF grating is written. This indicates that SFG experiences a more favorable AOP condition in comparison to SHG during the detuning change, leading to a lower AOP threshold and a competitive advantage. The overlayed dual-axis spectra in stage **ii** show that the SF signal is located between the FH and the first-order sideband at shorter wavelength (1044.9 nm). The noise measurement in Fig. 4e shows that the primary comb remains in a low-noise coherent state.

With detuning further approaching zero, the FH enters stages **iii** and **iv**, corresponding to MI comb states, where no upconverted signals can be observed. Figure 4d shows that the FH spectrum exhibits chaotic MI comb states^38,39^, associated with high intensity noise at the photodetector as shown in Fig. 4e. The absence of any upconverted signals can be due to unfavorable resonant condition at the SH band. It can also be ascribed to a disproportionate rise in photocurrent and photoconductivity within the high-power and high-phase-noise MI state, resulting in the pump power falling below the AOP threshold. Note that under different condition, and in the less conductive telecom range, 1550-nm MI combs can be upconverted under close to group-velocity matched condition between the FH and SH bands^40^.

The FH finally enters stage **v**, where a soliton crystal is observed, along with an upconverted signal, probably triggered by another low threshold doubly resonant condition being met. The overlayed spectrum in Fig. 4d shows that the FH comb exhibits a sech^2^-shaped envelope, in Fig. 4e that the FH goes back to a coherent low-noise state. During our experiment, the single soliton state was not observed as no specific power stabilization techniques were implemented^41^.

The combination of coherent comb generation and AOP as shown in Fig. 4 serves as the first direct experimental confirmation of self-assisted non-cascaded SFG. Achieving AOP in Si_3_N_4_ waveguides only requires the involving coherent lights exceeding the poling threshold, while in microresonators, a triply (or doubly for the case of degenerate processes) resonant condition is also necessary. Experimentally, both degenerate SFG (SHG), and cascaded SFG (effective THG) have been demonstrated^18^. In these cases, the AOP comes from the interaction of a pump and its direct harmonics. However, self-assisted non-cascaded SFG results from SF-AOP triggered by two coherent signals not related through harmonics.

In practice, such non-cascaded SF-AOP is constrained primarily by AOP competition. The presence of the two pumps can impede the SF AOP process if each of them can pole its own SH *χ*^(2)^ gratings. Notably, when the two beams are not identically strong, which is often the case, the stronger beam tends to dominate the competition resulting in regular SHG instead of SFG.

**Fig. 5:**
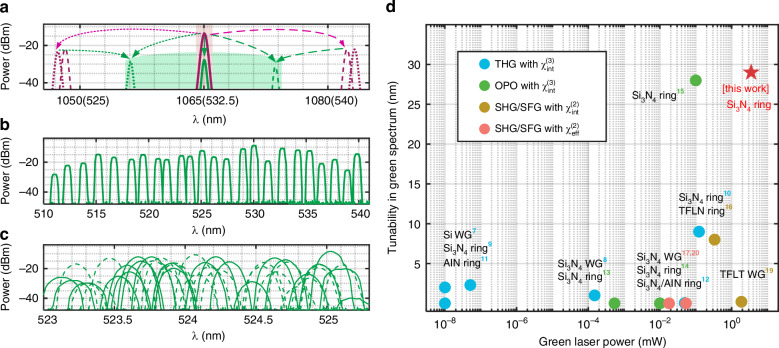
Performance of the green light source. **a** Overlapped spectra of the 11-nm-switchable green light generation and the associated 1-μm pump. Purple traces: the 1 μm band; green traces: the green band. Three different states are recorded by the OSA while pump within a single pump resonance, indicated by different line styles. Solid lines: SHG of the 1065 nm pump. Dotted line: primary comb generation and subsequent SFG between pump and blue detuning comb line. Dashed line: second primary comb state and subsequent SFG between pump and red detuned comb line. **b** Tunable range of 29 nm from 511 to 540 nm at a fixed temperature of 50 °C. **c** Green signals obtained in a 2-nm range at 50 °C (solid line) and 40 °C (dashed line). **d** Enhanced output power and spectral coverage compared to other integrated frequency-conversion green sources. WG: waveguide; TFLN: thin-film lithium niobate; TFLT: thin-film lithium tantalate

In our device, the non-cascaded coherent pump can be realized through Kerr combs generation by leveraging the anomalous dispersion at the pump, while the AOP threshold is significantly reduced to the mW level while maintaining small FSR. Given the presence of multiple comb lines with significant powers, the SF product can be switched depending on the achievable triply resonant condition. Figure 5a shows a typical case for switchable green light generation with a pump near 1065 nm. As the pump is slightly tuned, the green signals can be switched in a range of 11 nm as the primary comb state varies along with detuning conditions. The solid, dashed, and dotted traces in Fig. 5a represent the overlapped FH and green spectra recorded under three detuning conditions. Detailed spectra evolution with pump detuning can be found in Fig. S7.

When pump wavelength is varied in the range of 1030–1070 nm at a fixed temperature of 50 °C, a stacked display of multiple green signal spectra obtained via an OSA is plotted in Fig. 5b. The generated green light wavelength spans from 511 to 540 nm, distributed discretely throughout half of the green spectrum (510 to 560 nm). We observe a significant variation in the generated output power, which we attribute to the presence of different transverse modes participating in the AOP process, leading to varying outcoupling efficiencies, quality factors, and detuning conditions^29^. The interaction between the same pair of modes is observed repeatedly as a result of the Vernier effect, consistent with previous studies^18,29^. The green signal in Fig. 5b is selectively sampled to ensure that the interval is no more than 2 nm, but in fact, the green signal can be much denser. To demonstrate the density of the green signals, Fig. 5c shows all reconfigurable green signals obtained in a 2-nm span by varying pump wavelength under two fixed temperature. The pump exhibits a thermal coefficient of 14.2 pm/K, which can be used as a probing mechanism for precise detuning control. We measure a signal density of 10.5 nm^−1^ at 50 °C and 11.6 nm^−1^ at 40 °C, which is more than 6 times denser than OPO-based tunable green source 1.6 nm^−1^^15^.

## Discussion

Figure 5d summarizes the best performance metrics of on-chip green laser sources in terms of power and tunability in the green spectrum. Compared to previous studies, both the 3.5-mW green laser power (achieved in the 494-nm-gap device) and the 29-nm tunable span (achieved in the 567-nm-gap device) show improved performance. The high spectrum density of generated green light also allows for flexible frequency tuning in our device.

It is also important to note that our results were achieved using similar devices, with variations only in the couplers. This indicates that the benefits of high-power operation, wide tunability, and low AOP threshold demonstrated in this study can be integrated into a single device, provided that the coupling is appropriately optimized for both pump and green bands. To improve the performance of the demonstrated devices, one key approach is to realize critically coupled conditions at both pump and green band. Geometries such as asymmetric add-drops^28^ or pulley couplers^42^ can be implemented to achieve critical coupling in a single resonator at both FH and SH wavelengths. Improved coupling but also the expansion of the high-power operational range, can be realized in linearly uncoupled microresonators, which enable independent tuning of FH and SH resonances^40^. Consequently, an optimized doubly resonant condition can be replicated, in principle, across any wavelength within the pump tunable range.

Based on our theoretical modeling of AOP in resonant systems, the AOP threshold is inversely proportional to the microresonator finesse^30^. Despite reaching a mW-level AOP threshold, there is still substantial potential to reduce the AOP power by over an order of magnitude. Our microresonator, with a 50-GHz FSR and non-critical coupling, suggests that a microring with a 1-THz FSR could achieve an AOP threshold below 1 mW. Such ultralow threshold showcases the potential of this approach for developing a fully integrated self-injection-locked green laser source.

Additionally, by combining AOP with intrinsic *χ*^(3)^ processes, frequency-comb mediated non-cascaded SFG offers additional capability for wavelength tuning of the green light signal within a fixed pump resonance, opening up new possibilities for applications such as self-reference-stabilized comb. In this work, we demonstrate the mode pairs with angular frequencies *ω* and *ω* + Δ can achieve SF AOP. Analytically, higher-order mode pairs with frequencies *ω* − *m*Δ and *ω* + (*m* + 1)Δ (namely m^th^-order mode pairs) can also achieve AOP and SFG processes and contribute to the same SF frequency, with the phase relationships between different mode pairs being influenced by dispersion characteristics. Based on the primary combs we generated, the AOP and SFG contributions are predominantly from fundamental (0^th^-order) mode pairs. Detailed analysis can be found in Supplementary Note 5.

In summary, we have developed an integrated green source through photo-induced second-order nonlinearities, achieving record performance in terms of power and tunability. This work significantly enhances the capabilities of Si_3_N_4_ for visible light generation. From a physical standpoint, we provide the experimental evidence linking optical frequency comb to AOP conditions, demonstrate the feasibility of non-cascaded SF AOP for the first time, and utilize this approach to access a broader green light spectrum.

*Note:* During the peer review of this work, another example of green light generation in silicon nitride microring through SHG was reported in^43^.

## Methods

### TPM imaging of *χ*^(2)^ gratings

To characterize the gratings formed in Si_3_N_4_ waveguides, a 1010-nm femtosecond laser was focused on the grating plane of the microresonators. The laser focus was scanned across the grating plane while monitoring the SHG, enabling the detection of the *χ*^(2)^ response. Intrinsically, the centrosymmetric Si_3_N_4_ exhibits negligible SH response. However, at the locations of AOP-induced gratings, the built-in electric field generates an effective *χ*^(2)^, resulting in a detectable response that shows the period and further the phase mismatch.

## Supplementary information

Supplementary Information for: Integrated tunable green light source on silicon nitride

## Data Availability

The code used to produce the results of this paper are available at 10.5281/zenodo.17535321.
