# Supplementary material for: Integrated tunable green light source on silicon nitride
Source: Light Sci Appl. 2026 Feb 28;15:132. doi: 10.1038/s41377-026-02222-8 (PMC12949982; doi:10.1038/s41377-026-02222-8)
Supplement: Supplementary file 1 — Supplementary Information for: Integrated tunable green light source on silicon nitride [file 41377_2026_2222_MOESM1_ESM.pdf]

Supplementary Information for:
Integrated tunable green light source on silicon nitride

Gang Wang,[1] Ozan Yakar,[1] Xinru Ji,[2] Marco Clementi,[1,3] Ji Zhou,[1] Christian Lafforgue,[1] Jiaye Wu,[1] Jianqi Hu,[2] Tobias J. Kippenberg,[2] and Camille-Sophie Brès[1,*]

[1] *École Polytechnique Fédérale de Lausanne, Photonic Systems Laboratory (PHOSL), Lausanne, Switzerland*
[2] *École Polytechnique Fédérale de Lausanne, Laboratory of Photonics and Quantum Measurements (LPQM), Lausanne, Switzerland*
[3] *Dipartimento di Fisica "A. Volta", Università di Pavia, Via A. Bassi 6, 27100 Pavia, Italy*

Supplementary Note 1. Si_3N_4 microresonator devices

The Si_3N_4 microresonators used in this study have a racetrack geometry, coupled to a bus waveguide via a directional coupler, with design parameters shown in Fig. S1a. Two different coupler gaps 494 and 567 nm are used with other parameters remaining the same. Both the microresonator waveguide and the bus waveguide have cross-sectional dimensions of 1.3×0.9 μm^2. In this geometry, the simulated effective index (n_{eff}), free spectral range (FSR), and dispersion parameter (D) of the transverse electric (TE) modes at fundamental harmonic (FH) and second harmonic (SH) are illustrated in Figs. S1b-S1d. Notably, the fundamental TE mode exhibits anomalous dispersion ($D > 0$) at wavelengths longer than 970 nm, enabling comb generation through third-order nonlinearity. The TE transverse mode profiles of SH1-4 are depicted in Figs. S1e-S1h. These mode profiles and their effective refractive indices are subsequently used to simulate the theoretical pattern of the optically inscribed gratings.

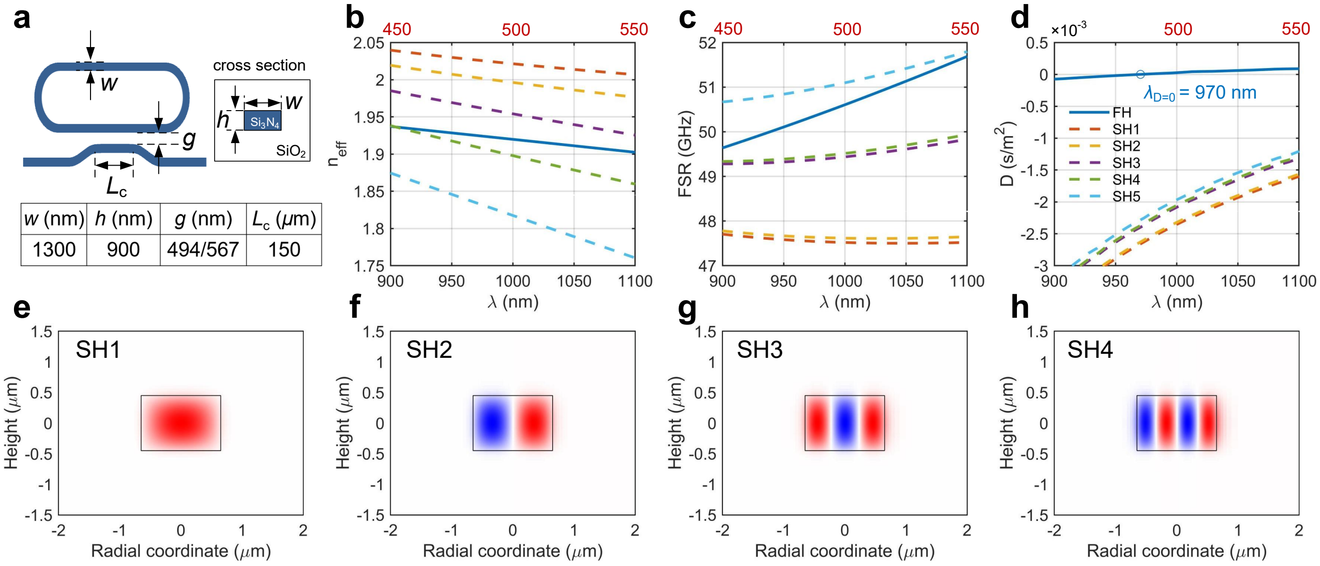

Figure S1. **Design and simulation of the Si_3N_4 microresonators. a** Schematic of the Si_3N_4 racetrack microresonator. The racetrack microresonator is coupled to the bus waveguide with a directional coupler. The parameters are listed on the table. w: width; h: height; g: gap; L_c: coupler length. **b-d** Simulated effective refractive index, FSR, and dispersion parameter D of fundamental mode at FH wavelength and first five SH (SH1-SH5) modes. **e-h** Simulated TE-polarized mode amplitude distributions of SH1-SH4.

Figure S2a shows the setup for characterizing resonance linewidth using the sideband modulation technique[1]. The laser output is polarization-maintaining and tunable within 1030–1070 nm. The first polarization controller is added to set the polarization state to TM, which is required for phase modulation in the electro-optic modulator (EOM). The microwave generator gives a 2-GHz signal to the EOM, which creates two sidebands as reference to calibrate the resonance sweep. The laser is then set to TE polarization and coupled into the bus waveguide through a free-space collimator and a lens. The output laser is collected by a lens and sent to a photodetector connected to the oscilloscope.

Figures S2b and c show the resonance linewidth at 1065-nm of two resonators with 567-nm and 494-nm gap, respectively. The loaded Q factors Q_L of the two resonators are 1.78×10^6 and 0.82×10^6, as well as the minimum transmittance $T_{\min}$ are 0.56 and 0.76 respectively. To calculate the intrinsic and coupling Q factor Q_i and Q_c, we use the equation:

$$Q_i, Q_c = \frac{2Q_L}{1 \pm \sqrt{T_{\min}}} \quad (1)$$

The $\pm$ in the denominator of the formula depends on the coupling condition to the microresonator: if over-coupled $Q_i > Q_c$, and if under-coupled $Q_i < Q_c$. For the microresonator with 567-nm gap, we have $Q_{i1}, Q_{c1} = 2.03 \times 10^6, 14.27 \times 10^6$. Finite-difference time-domain (FDTD) simulations of the coupler suggest the coupling Q should be approximately 2.53×10^6. Since the measured value of 2.03×10^6 closely aligns with the simulated coupling Q, we conclude that 2.03×10^6 represents the coupling Q, while 14.27×10^6 corresponds to the intrinsic Q. Similarly, we have $Q_{i2}, Q_{c2} = 0.87 \times 10^6, 12.77 \times 10^6$ for the second microresonator with 494-nm gap. Based on the FDTD

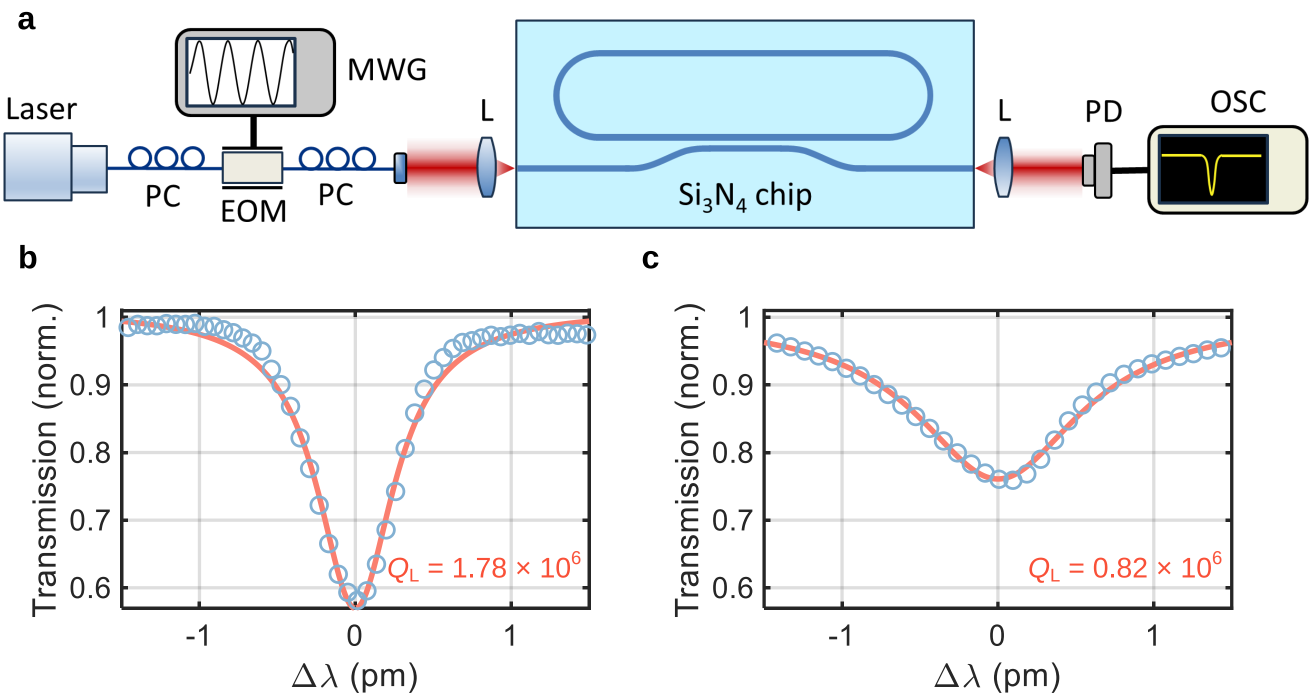

Figure S2. **Setup and characterization of the Si_3N_4 microresonators.** **a** Experimental setup for characterizing the resonance linewidth of microresonators. PC: polarization controller; EOM: electro-optic modulator; MWG: microwave generator; C: collimator; L: lens; PD: photodetector; OSC: oscilloscope. Resonance linewidth measurement of **b** the 567-nm and **c** 494-nm gap microresonator devices. The measured linewidths (blue points) are fitted with a Lorentzian function (red curve), showing the loaded Q factor (Q_L) of 1.78×10^6 and 0.82×10^6, respectively.

simulation, the coupling Q should be approximately 0.98×10^6, leading to the conclusion that 0.87 M is the coupling Q and 12.77×10^6 is the intrinsic Q. This conclusion is also supported by a comparison between the two devices, which differ only in their coupler gaps. Since their intrinsic Q values are expected to be similar, the proximity of 12.77×10^6 and 14.27×10^6 reinforces the conclusion that these are the respective intrinsic Q for the devices. Comparing with the measured Q_i, Q_c,we can tell the two microresonators are both over-coupled at 1 µm region, meaning the coupling losses exceed the intrinsic losses of the microresonators. However, for SH fundamental transverse mode, the directional coupler only couples less than 0.01% of green light, together with the large intrinsic loss, resulting in significantly under-coupled condition. The device with the narrower gap exhibits approximately an 8-dB improved out-coupling rate of the generated green light, although for a 1-µm pump, it tends to be over-coupled with reduced field enhancement. In contrast, the broader-gap device approaches critical coupling for the pump, facilitating low-power all-optical poling (AOP) and comb generation, though it offers lower extraction efficiency for the green light. The calculated field intensity enhancement factors F for the two devices at the pump wavelength are 88.2 and 143.0, respectively, according to $F = 2\lambda Q_L^2/\pi n_g L Q_c$, where λ is wavelength, Q_L is the loaded quality factor, n_g is group index, L is cavity length, and Q_c is the coupling quality factor. In the current demonstration, the narrower-gap device is employed for high-power green light generation, where efficient extraction of green light is the primary objective, while the broader-gap device is used for low-power, comb-mediated AOP, where the primary goal is maximizing the intensity enhancement at the pump wavelength.

Supplementary Note 2. Experimental setup

The setup for AOP and detection is shown in Fig. S3. Compared to the linewidth measurement setup in Fig. S3a, the EOM here is much weakly driven by the vector network analyzer (VNA) that sends 50–1500 MHz signal. The VNA then receives the response of SH to measure the transfer function. An ytterbium-doped fiber amplifier (YDFA) is inserted after the EMO. The FH laser (red arrow) is coupled to the ring and interacts with the defect-induced weak SH to generate periodic photocurrent in the waveguide to pole an intracavity grating, which in turn enhance the SH through positive feedback. The generated SH is then coupled out of the ring through the directional coupler and

emitted from the facet of the chip. A shortpass dichroic mirror is used to separate FH from SH, and additional filters in the SH path can be used to remove any residual FH. Both FH and SH are collected by two collimators and sent to different photodetectors and instruments to measure their power, spectra, and transfer function. The resolution of infrared and visible optical spectrum analyzers (OSAs) are set to 0.5 and 0.1 nm, respectively. The taper loss is measured to be 3.3 dB by comparing the measured Fabry–Pérot interference with the theoretical case without any taper loss. The loss of the C-coating lens, dichroic mirror, and filters is measured to be 3.1 dB in total for SH.

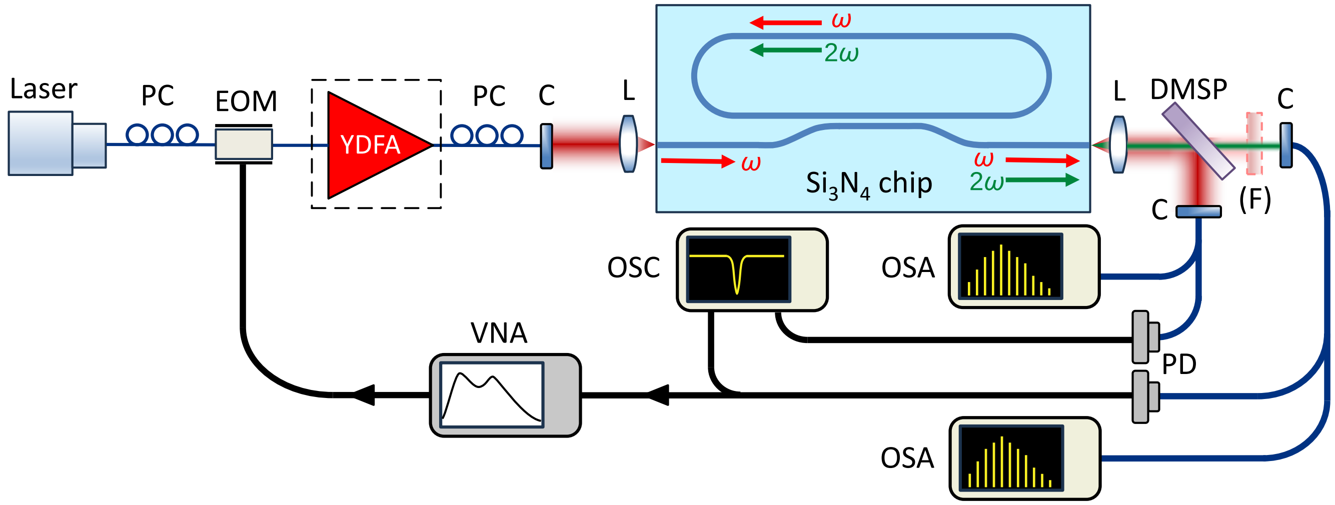

Figure S3. **Experimental setup for all-optical poling, measuring, and probing the SH response.** A YDFA is added after the EOM. The dashed box represents that the YDFA is not used in some of the experiments. A short-pass dichroic mirror (DMSP) is added after the outcoupling lens to separate FH and SH signal. The FH signal leaks a small proportion through the DMSP and can be filtered out completely using several low-pass filters (F). Two collimators collect the output FH and SH separately and send them to the oscilloscope (OSC) and OSAs. A VNA is used to probe the FH and SH resonance through sending a weak modulation signal (50-1500 MHz) to the EOM and detecting the RF response of the SH.

Supplementary Note 3. Fine scan of reconfigurable AOP and $\chi^{(2)}$ measurement

To show the broadband SHG and the identification of mode pair interactions of the 494-nm-gap device, we carry a finer TE polarized scan in the range of 1060–1070 nm region for fixed pump power and temperature same as Fig. 2e. Figure S4a shows the on-chip SH power during pump wavelength scan. Within all the 62 fundamental FH resonances, 39 of them gives reconfigurable SH response. We primarily focused on high-power AOP working points exceeding 1 mW, and selected several resonances of interest to do two-photon microscopy (TPM), where the typical TPM patterns observed (FH–SH1 to FH–SH4) are shown in Fig. S4b–S4e and demonstrate strong agreement with simulations of inscribed $\chi^{(2)}$ gratings.

We estimate the intracavity $\chi^{(2)}_{\text{eff}}$ by comparing the TPM response of a poled ring to that of a waveguide under the same TPM parameters including laser power, wavelength, focus, etc. The intensity of TPM response is proportional to the square of $\chi^{(2)}_{\text{eff}}$. Figures S5a and S5b show the TPM pattern of the poled waveguide and the averaged response over distance in the focused area, respectively. Similarly, the maximum TPM response of the poled ring is shown in Figs. S5c and S5d. The response of the resonator is 1.51 times that of the waveguide, which means that $\chi^{(2)}_{\text{eff}}$ of the ring is 1.23 times $\chi^{(2)}_{\text{eff}}$ in the waveguide. The $\chi^{(2)}_{\text{eff}}$ of the poled waveguide is estimated to be 0.024 pm/V from its conversion efficiency[2]. Therefore the intracavity $\chi^{(2)}_{\text{eff}}$ is approximately 0.03 pm/V.

Supplementary Note 4. Additional proofs of sum-frequency all-optical poling

Figure S6 shows the observation of sum-frequency generation (SFG) during a slow resonance scan at 1065 nm and 1045 nm respectively. The top patterns show the evolution of FH from CW to a series of primary combs based on

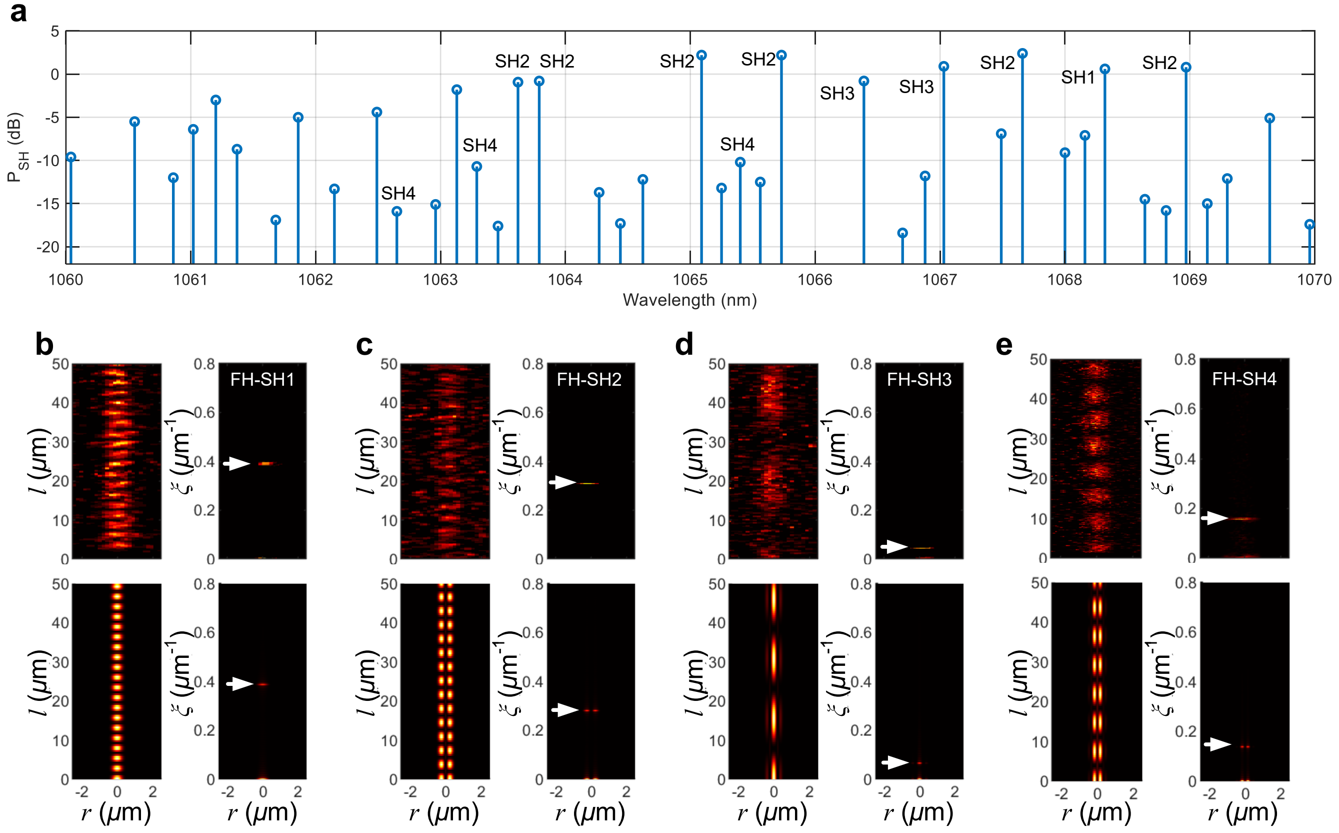

Figure S4. **TE polarization resonance scan with reconfigurable intracavity gratings.** **a** Generated SH power during the wide scan of pump from 1060 to 1070 nm, where 63% of the resonances can realize AOP. **b-e** Measured TPM gratings distribution (top left) and their spatial period (top right), as well as the simulation counterpart (bottom).

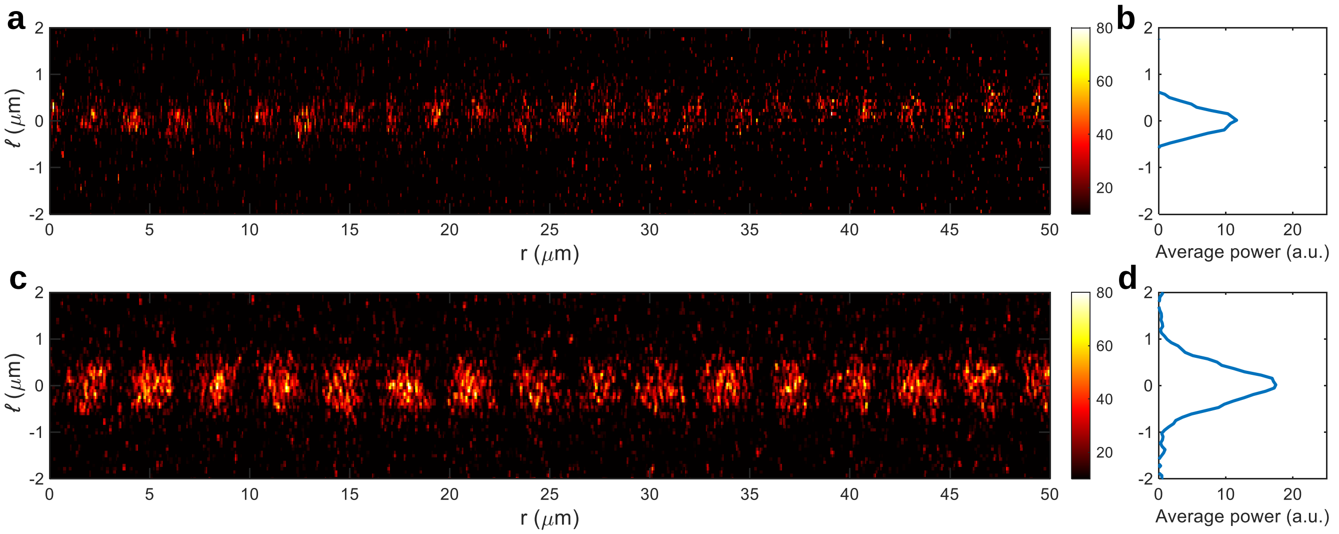

Figure S5. **Intracavity $\chi^{(2)}_{\text{eff}}$ measurement.** **a** and **c** TPM response of a poled waveguide and the poled microresonator. **b** and **d** show the averaged TPM response of **a** and **c** over distance.

detuning conditions. The bottom patterns show the evolution of SH/SF spectrum, with the dashed white line marking the half wavelength of the FH pump. Any possible gratings before the measurement were bleached.

After the generation of certain primary combs, SF signals are observed approximately 5 nm away from half of FH

wavelength. The wavelengths of the SF signal match well with a quarter of the sum of the pump and a combline. Notably, previous studies have reported SFG driven by SHG gratings, but the SFG observed here originates from self-induced AOP gratings supported by several reasons: (i) No SHG signal can be detected during the resonance sweep, ruling out the existence of SH-related gratings; (ii) If SHG gratings were present, their QPM bandwidth would be limited to approximately $\delta\lambda_{\mathrm{FWHM}} \approx 0.44\lambda_{\mathrm{FH}}^2/((n_{\mathrm{g}}(\lambda_{\mathrm{SH}}) - n_{\mathrm{g}}(\lambda_{\mathrm{FH}}))L_{\mathrm{eff}}) = 0.04$ nm, where n_{g} is the group index, L_{eff} is the effective waveguide length calculated by $L_{\mathrm{eff}} = c\tau_{\mathrm{p}}/n_{\mathrm{eff}} = cQ_{\mathrm{L}}/n_{\mathrm{eff}}\omega_0 = 157$ mm. The observed SFG signal, however, is separated by about 5 nm from the SH central wavelength, far beyond the range of SH gratings.

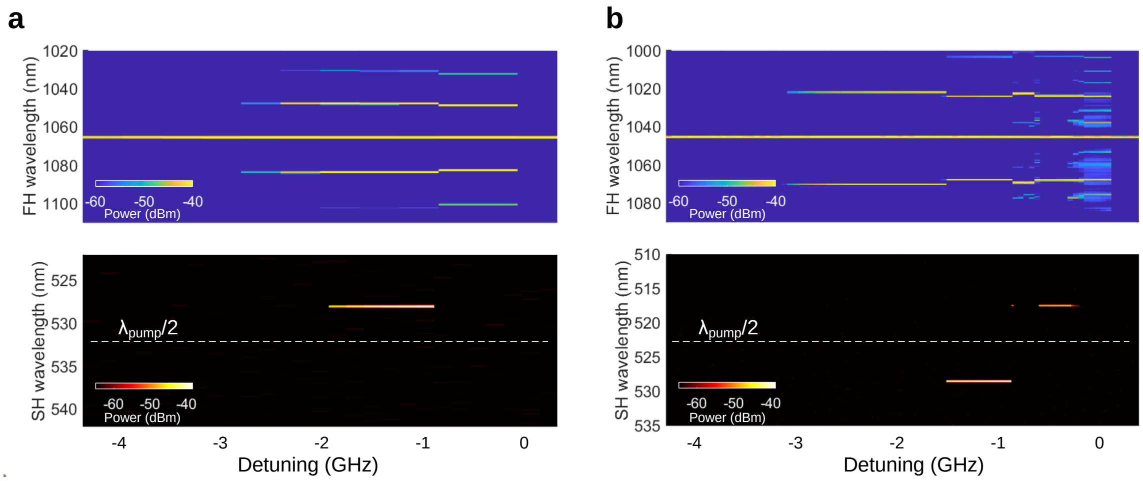

Figure S6. **Sum-frequency AOP without SHG. a and b** Evolution of FH (top) and SF (bottom) spectra in single linear resonance sweep at 1065 and 1045 nm, respectively. When doubly resonant condition between SF and SH is not met, the triply resonant condition between FH, primary comb sideband, and SF can be realized, which enables the SF AOP. These results further demonstrate that SH and SF generation depend on distinct grating structures, making them incapable of supporting each other's generation in our device.

In addition to realizing solely SFG within a single resonance scan, it is also possible to alternate between SHG and SFG by controlling the temperature to meet both doubly and triply resonant conditions. The SH/SF wavelength span under the fixed pump wavelength can reach up to 11 nm.

Figure S7 illustrates the transition of second-order frequency transition signals from SH to SF1 and SF2. Figures S7a and S7b depict the spectra of FH and SH/SF, where the SH signal gradually intensifies in the CW state but diminishes as the primary comb emerges. Correspondingly, when the triply resonant condition is met, SF1 signal rapidly occurs under strong coherent pump and disappears promptly after the primary comb transitions. The newly generated primary comb subsequently rewrites the grating, leading to the formation of the SF2 signal. Figure S7d shows the corresponding spectra of FH and SH/SF at three different stages.

Figure S7c illustrates the evolution of the VNA response, which exclusively detects SH/SF signals. Consequently, no response can be observed in regions where SH or SF are not generated. When SH is generated, the VNA detects a single peak that gradually shifts toward zero frequency (zero detuning). This peak corresponds to the FH detuning, indicating that FH approaches the effective resonance frequency. Due to dominant intrinsic absorption at visable band, the resonance peak of SH/SF cannot be detected.

In contrast to the single-peak SH case, when SF is generated, two resonance peaks are observed, marked by the dashed lines in Figs. S7c and S7e. Figure S7e provides a fine VNA measurement during SFG generation. The position of the left resonance peak remains nearly constant, corresponding to the thermally locked FH signal. The right resonance peak gradually shifts toward zero detuning, corresponding to the sideband of the primary comb. Due to limitations in the VNA scanning speed, the SF2 signal is not detected.

Supplementary Note 5. Multi-pairs-involved sum-frequency all-optical poling

In the main text, when discussing non-cascaded SF AOP, the primary focus is on adjacent mode pairs with angular frequencies ω and $\omega + \Delta$. This section further examines the contributions from higher-order mode pairs with angular frequencies $\omega - m\Delta$ and $\omega + (m+1)\Delta$ (i.e., the mth-order mode pairs, m is the integer index).

Regarding AOP, the electric field amplitude of the grating generated via the coherent photogalvanic effect is proportional to the photocurrent j_{ph}, which can be expressed as[2]

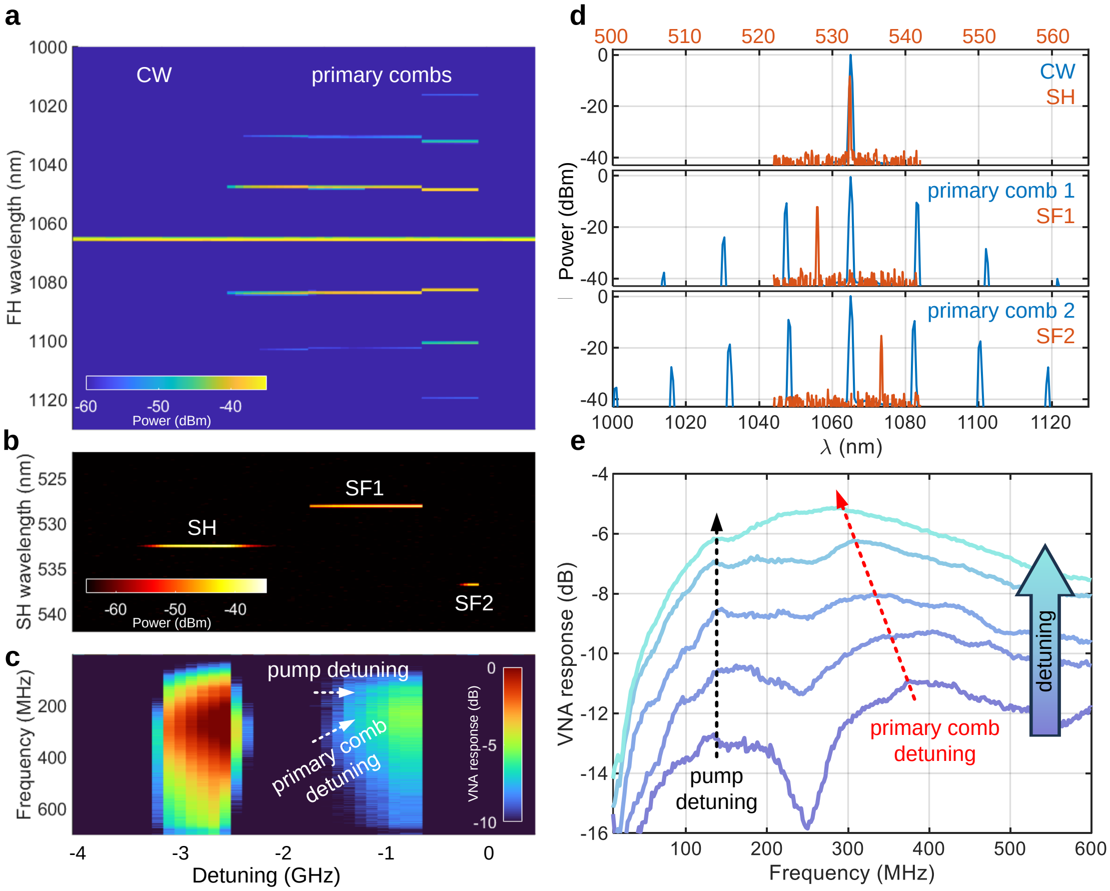

Figure S7. **Realization and detection of self-assisted SFG. a-c** Evolution of FH, SH/SF, and VNA response in a resonance sweep. FH experiences a CW stage and two primary comb stages. SH is first detected and then replaced by SF in primary comb stages. The VNA response shows single peak at CW stage and shows a dual-peak characteristic in the primary comb stage. **d** Overlayed dual-axis spectra of FH and corresponding SH/SF. Two self-assisted SF signals with the wavelength difference of 11 nm are realized. **e** Fine VNA sweep of the SFG generation. The left peak corresponds to FH detuning and the right one reflects the sideband detuning.

$$j_{\mathrm{ph}} = \sum_m j_{\mathrm{ph}}^{(m)} = \sum_m \left[\beta E_{2\omega+\Delta} E^*_{\omega-m\Delta} E^*_{\omega+(m+1)\Delta} + \mathrm{c.c.}\right] \tag{2}$$

where $j_{\mathrm{ph}}^{(m)}$ represents the photocurrent contributed by the mth-order mode pairs, β is the photogalvanic coefficient, $E_n = A_n e^{ik_n z}$ is the electric field, and A_n and k_n are the amplitude and wave vector of E_n, z is propagation distance.

When considering Taylor expansion to quadratic terms, the wave vector of $j_{\mathrm{ph}}^{(m)}$ can be written as:

$$k_{\mathrm{j}}^{(m)} = k_{2\omega+\Delta} - \left[k_{\omega-m\Delta} + k_{\omega+(m+1)\Delta}\right] = \delta k + \delta v_{\mathrm{g}}^{-1}\,\Delta + \frac{1}{2}\delta\beta_2\,\Delta^2 - m(m+1)\beta_{2,\omega}\,\Delta^2 \tag{3}$$

where $\delta k = k_{2\omega} - 2k_{\omega}$ is the mean momentum mismatch, $\delta v_{\mathrm{g}}^{-1} = v_{\mathrm{g},2\omega}^{-1} - v_{\mathrm{g},\omega}^{-1}$ is the inverse group velocity mismatch, $v_{\mathrm{g},\omega}(v_{\mathrm{g},2\omega})$ is the group velocity of light at $\omega(2\omega)$ frequency, and $\delta\beta_2 = \beta_{2,2\omega} - \beta_{2,\omega}$ is the group velocity dispersion mismatch, $\beta_{2,\omega}(\beta_{2,2\omega})$ is the second-order dispersion coefficient at $\omega(2\omega)$ frequency. Note that nonlinearity is neglected in our case, as the intensity of primary combs is much lower than the dispersion–nonlinearity-balanced soliton.

Given the fixed phase relationship among the comb lines of a primary comb[3], the wavevector mismatch can reflect the relationship of different photocurrent contributions. We examine the mismatch between the mth-order and fundamental wavevector:

$$k_{\mathrm{j}}^{(m)} - k_{\mathrm{j}}^{(0)} = -m(m+1)\,\beta_2\,\Delta^2 \tag{4}$$

We can further calculate the phase mismatch between the mth-order and fundamental mode pairs' contribution, that is, $\delta\phi = -m(m+1)\,\beta_2\,\Delta^2\,L_{\text{eff}}/2$, where L_{eff} is the effective waveguide length. This indicates that phase mismatch increases nearly quadratically with the mode pair order m. However, even when we consider the 4th-order mode pairs that are approximately 180 nm away from each other, they only show a phase mismatch of less than 0.4π, which means most of our comb lines can contribute to the same photogalvanic field constructively.

Practically, given that the power difference between each comb sideband is approximately 10 dB (corresponding to a 5 dB difference in electric field amplitude), the photocurrent contribution from m^{th}-order mode pairs is expected to be 10 dB higher than that of $(m+1)^{\text{th}}$-order mode pairs, assuming the photogalvanic coefficient β remains constant.

When analyzing SFG, we can assume a steady-state regime where the effective $\chi^{(2)}$ strength induced by optical parametric processes remains constant. According to the coupled wave equation:

$$\frac{\partial A_{\text{SF}}}{\partial z} = ig\sum_m A_{\omega-m\Delta}\,A_{\omega+(m+1)\Delta}\,e^{\,i\left[k_{\omega-m\Delta}+k_{\omega+(m+1)\Delta}-k_{2\omega+\Delta}\right]z} \tag{5}$$

where g is the coupling strength reflecting the magnitude of $\chi^{(2)}$. For simplicity, we neglect the resonance condition of the generated SFG signal, as it does not significantly affect the relative contributions of different mode pairs.

Similar to the AOP case, we examine the wavevector mismatch between m^{th}-order and fundamental mode pairs, which effectively is $-k_{\text{j}}^{(m)}$. This indicates that the relationship between different mode pair contributions in SFG mirrors that in the AOP process. Considering amplitudes, we again find that: $\frac{\left|A_{\omega-m\Delta}\,A_{\omega+(m+1)\Delta}\right|}{\left|A_{\omega-(m+1)\Delta}\,A_{\omega+(m+2)\Delta}\right|} \approx 10$. Therefore, the conclusion for SFG is consistent with that for AOP: various mode pairs contribute constructively in principle, while practically fundamental mode pairs dominate the process.

---

* camille.bres@epfl.ch

[1] Li, J., Lee, H., Yang, K. Y. & Vahala, K. J. Sideband spectroscopy and dispersion measurement in microcavities. *Optics Express* **20**, 26337–26344 (2012).

[2] Yakar, O., Nitiss, E., Hu, J. & Brès, C.-S. Generalized coherent photogalvanic effect in coherently seeded waveguides. *Laser & Photonics Reviews* **16**, 2200294 (2022).

[3] Coillet, A. & Chembo, Y. On the robustness of phase locking in kerr optical frequency combs. *Optics Letters* **39**, 1529–1532 (2014).
